# Influence of ovarian stromal cells on human ovarian follicle growth in a 3D environment

**DOI:** 10.1093/hropen/hoad052

**Published:** 2023-12-21

**Authors:** Monika Grubliauskaitė, Hanne Vlieghe, Saeid Moghassemi, Arezoo Dadashzadeh, Alessandra Camboni, Živilė Gudlevičienė, Christiani A Amorim

**Affiliations:** Institute of Biochemistry, Life Sciences Center, Vilnius University, Vilnius, Lithuania; Department of Biobank, National Cancer Institute, Vilnius, Lithuania; Pôle de Recherche en Physiopathologie de la Reproduction, Institut de Recherche Expérimentale et Clinique, Université Catholique de Louvain, Cliniques Universitaires Saint-Luc, Brussels, Belgium; Pôle de Recherche en Physiopathologie de la Reproduction, Institut de Recherche Expérimentale et Clinique, Université Catholique de Louvain, Cliniques Universitaires Saint-Luc, Brussels, Belgium; Pôle de Recherche en Physiopathologie de la Reproduction, Institut de Recherche Expérimentale et Clinique, Université Catholique de Louvain, Cliniques Universitaires Saint-Luc, Brussels, Belgium; Pôle de Recherche en Gynécologie, Institut de Recherche Expérimentale et Clinique, Université Catholique de Louvain, Cliniques Universitaires Saint-Luc, Brussels, Belgium; Anatomopathology Department, Cliniques Universitaires Saint-Luc, Brussels, Belgium; Faculty of Medicine, Vilnius University, Vilnius, Lithuania; Pôle de Recherche en Physiopathologie de la Reproduction, Institut de Recherche Expérimentale et Clinique, Université Catholique de Louvain, Cliniques Universitaires Saint-Luc, Brussels, Belgium

**Keywords:** ovarian follicle, 3D culture environment, stromal cell, artificial ovary, fertility preservation, co-culture, follicle development

## Abstract

**STUDY QUESTION:**

Do ovarian stromal cells (OSCs) influence the viability and growth of human preantral follicles *in vitro*?

**SUMMARY ANSWER:**

A feeder layer of OSCs promotes the growth and transition of low developmental stage follicles to the primary/secondary stage while maintaining a high proportion of viable follicles.

**WHAT IS KNOWN ALREADY:**

In the ovary, follicles rely on the support of ovarian cells, which secrete essential factors for their survival and development. This phenomenon has also been demonstrated *in vitro* through the 3D culture of isolated mouse primary and secondary follicles on a feeder layer of OSCs. This co-culture notably enhances follicle survival and growth.

**STUDY DESIGN, SIZE, DURATION:**

Pre-antral follicles were isolated from human frozen-thawed ovarian tissue biopsies and then encapsulated in 1% alginate scaffolds. These embedded preantral follicles were either placed directly on the OSCs feeder layer or at the bottom of a culture dish for a 7-day *in vitro* culture (control). The study compared follicle viability, growth, and hormone production between the different groups.

**PARTICIPANTS/MATERIALS, SETTING, METHODS:**

Primordial/intermediate and primary follicles were isolated from frozen-thawed ovarian tissue of cancer patients (n = 6). OSCs were then isolated from ovarian tissue of post-menopausal women and cultured as a feeder layer. Follicle diameter was measured on Days 0 and 7 using an inverted microscope to assess their development based on the increase in diameter. Viability was evaluated by staining a subset of follicles (n = 87) with calcein AM and ethidium homodimer-I, followed by classification into healthy/minimally damaged and damaged/dead follicles using confocal fluorescence microscopy. Additionally, estradiol levels were measured using ELISA.

**MAIN RESULTS AND THE ROLE OF CHANCE:**

A total of 382 human preantral follicles (370 primordial/intermediate and 12 primary) with a mean diameter of 40.8 ± 9.9 µm (mean ± SD) were isolated, embedded in 1% alginate hydrogel, and placed either on a monolayer of OSCs or directly on the plastic. By Day 7, the preantral follicles showed a significant size increase under both culture conditions (*P* < 0.0001 for D0 vs D7). The mean diameter of follicles (quiescent and growing) cultured on the feeder layer was 80.6 ± 11.0 μm compared to 67.3 ± 7.2 μm without it (*P* = 0.07). During the 7-day *in vitro* culture, the viability of the follicles significantly decreased only in the group without an OSCs monolayer compared to the D0 viability (*P* < 0.05). Additionally, more follicles transitioned to a higher developmental stage in the presence of OSCs (D0 primordial/intermediate: 184, primary: 7 vs D7 primordial/intermediate: 51, primary/secondary: 93) compared to those cultured without OSCs (D0 primordial/intermediate: 186, primary: 5 vs D7 primordial/intermediate: 84, primary/secondary: 65; *P* < 0.001). Specifically, 66 and 44 follicles reached the secondary stage (75< x <200 μm) in the presence and absence of OSCs, respectively. Moreover, the estradiol level was significantly higher (*P* = 0.006) in the alginate beads containing primordial and growing follicles cultured on the OSCs (54.1 ± 14.2 pg/ml) compared to those cultured without OSCs (29.9 ± 4.0 pg/ml).

**LARGE SCALE DATA:**

N/A.

**LIMITATIONS, REASONS FOR CAUTION:**

This study was conducted using a short-term culture, and none of the primordial/intermediate/primary follicles reached the antral stage. Further *in vitro* studies are required to investigate follicular developmental capacity, physiology, and steroidogenesis in alginate scaffolds with human OSCs.

**WIDER IMPLICATIONS OF THE FINDINGS:**

Activating and growing human primordial/intermediate follicles to a secondary stage in *in vitro* short-term culture has posed a longstanding challenge. However, co-culturing with human OSCs has shown the potential to overcome this limitation.

**STUDY FUNDING/COMPETING INTEREST(S):**

This study was supported by grants from the Fonds National de la Recherche Scientifique de Belgique (FNRS-PDR Convention grant number T.0004.20 awarded to C.A.A., PhD scholarship awarded to H.V.), Fondation Louvain (awarded to C.A.A.; PhD scholarship awarded to S.M., as part of a legacy from Mr Frans Heyes, and PhD scholarship awarded to A.D. as part of a legacy from Mrs Ilse Schirmer), Foundation Against Cancer (grant 2018-042 awarded to A.C.), and the European Community Structural Funds and Lithuanian Research Council (Agreement registration No. D-19-0874). The authors have no conflicts of interest to declare.

WHAT DOES THIS MEAN FOR PATIENTS? Ovarian tissue cryopreservation can be the sole option to preserve fertility and endocrine function for prepubertal girls or reproductive-age patients who cannot delay cancer treatment but still wish to have the ability to bear genetically related children. However, cryopreservation and reimplantation of ovarian tissue during remission are not feasible for patients with hematological malignancies due to the high risk of cancer cell recurrence within the ovarian tissue. One potential solution to overcome this limitation could involve creating an artificial ovary using ovarian follicles isolated from cryopreserved ovarian tissue and other ovarian tissue cells from the patients when in remission. Successful artificial ovary models have been achieved in rodents, and the significance of ovarian cells in follicle development has been emphasized. Our study aimed to investigate the impact of human ovarian cells from post-menopausal patients on the survival and development of isolated human dormant and growing follicles. Our findings revealed that the ovarian cells positively influence follicle growth, quality, development, and hormone secretion *in vitro*. These results suggest that our approach may have significant implications for enhancing assisted reproductive technologies and fertility preservation strategies for cancer patients.

## Introduction

Ovaries are responsible for hormone production and gamete generation in women. However, these functions can be disrupted by oncological treatment. Therefore, ovarian tissue cryopreservation can be the only option to preserve fertility and endocrine function for prepubertal girls or reproductive-age patients who cannot delay gonadotoxic therapy (Practice Committee of the American Society for Reproductive Medicine, 2019). However, while transplantation of ovarian tissue during remission in cancer patients has resulted in pregnancies and healthy newborns worldwide (Andersen *et al.*, 2019; Sheshpari *et al.*, 2019), it harbors the risk of reintroducing malignant cells in some malignancies, such as leukemia (Practice Committee of the American Society for Reproductive Medicine, 2019). While isolated primordial follicles from the tissue minimize the risk of cancerous cell transmission, the lack of stromal cells affects follicle activation and growth *in vitro*.

Folliculogenesis is a complex process during which interactions between somatic cells and the oocyte are required (Simon *et al.*, 2020). For the primordial follicle to transition to the primary stage, granulosa cells around the oocyte have to start proliferating, releasing these signals from the ovarian somatic cells (Tagler *et al.*, 2011; Dolmans and Amorim, 2019; Simon *et al.*, 2020). Once activated, the follicles recruit surrounding cells for structural support and provide the precursors for estrogen synthesis (Dolmans and Amorim, 2019). Apart from these recruited cells, the ovarian cortex consists of diverse cell populations (e.g. stromal, immune, perivascular and endothelial cells) (Wagner *et al.*, 2020) that also play numerous roles which directly or indirectly affect the follicles and are therefore necessary in *in vitro* culture conditions. Indeed, a study on mouse preantral follicles revealed that using a feeder layer, consisting primarily of theca cells and macrophages (Tingen *et al.*, 2011) or mouse embryonic fibroblasts (Tagler *et al.*, 2012), during *in vitro* culture significantly improved follicle survival and growth. Beneficial effects of a peritoneum mesothelial stem cell feeder layer were also observed in a short-term *in vitro* culture of mouse preantral follicles as evidenced by significantly increased follicular growth, estradiol production, and a high eccentric follicular rate, while there was no impact on follicle viability (Sarabadani *et al.*, 2021). Based on these promising results in a rodent model, the goal of our study was to assess the effect of a feeder layer of ovarian stromal cells (OSCs) on the survival and development of isolated human preantral follicles. We hypothesize that stromal cell paracrine signaling positively influences follicle growth, quality, and activation *in vitro*.

## Materials and methods

### Experimental design

**Figure 1.**
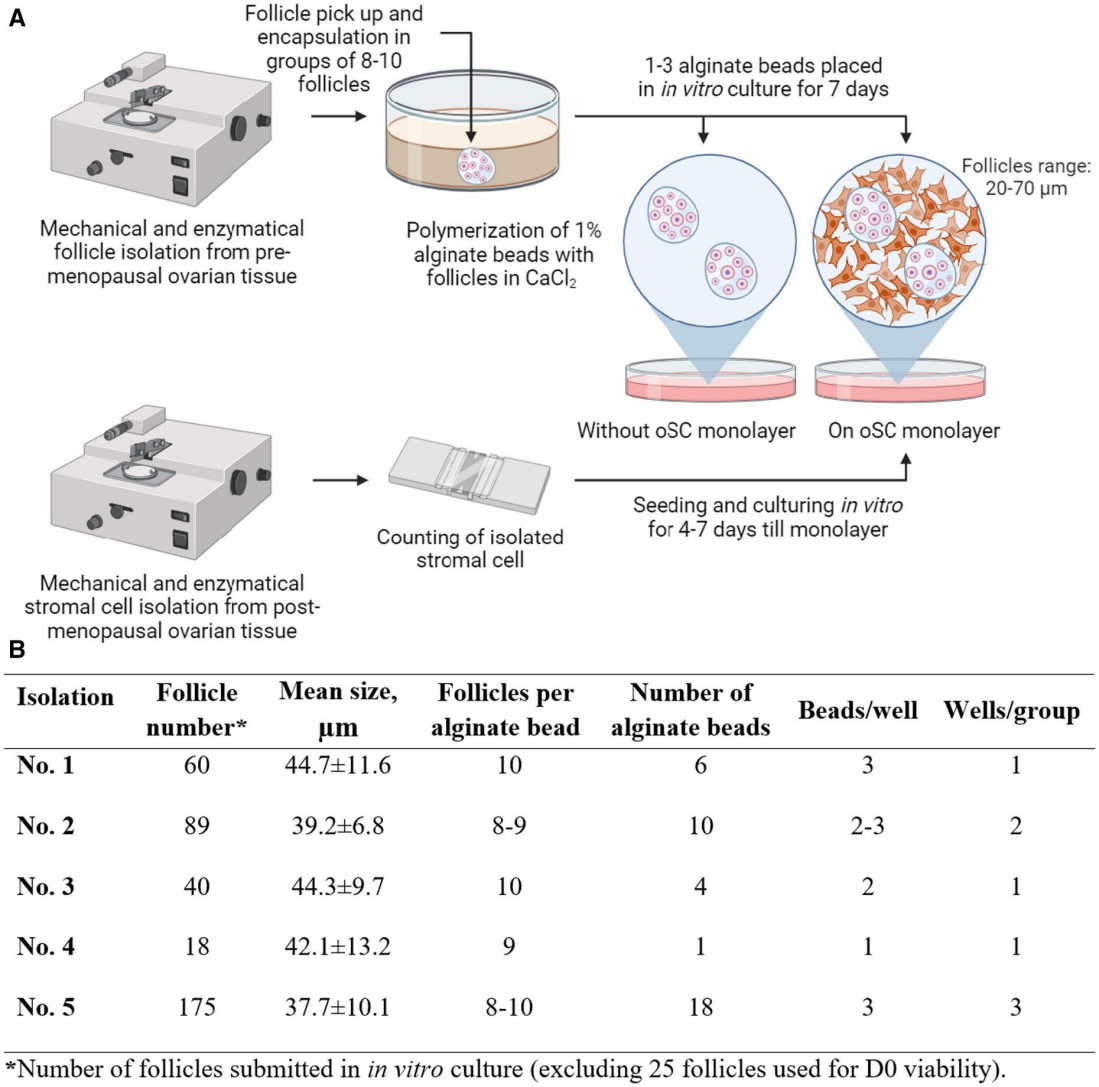
**Experimental preparation design.** (**A**) A schematic representation of the experimental workflow outlining the *in vitro* culture experiment. (**B**) Details regarding the encapsulation of follicles within alginate beads and their arrangement for subsequent *in vitro* culture. Scheme created using Biorender.com. oSC: ovarian stromal cells; D0: day zero.

OSCs were isolated from five multiorgan donor patients who had undergone menopause to establish a cell culture that closely mimics the environment found in the ovaries of cancer survivors, and the OSCs were allowed to grow *in vitro* to form a monolayer to act as the feeder layer. Preantral follicles were isolated from human frozen-thawed biopsies from six patients and embedded in a 1% alginate matrix. They were cultured *in vitro* for 7 days, either with or without the feeder layer of ovarian cells (Fig. 1A). Thereafter, the follicles were analyzed for survival and development, and the spent medium was assessed for hormone levels.

### Ethical approval

The Review Board of the Université Catholique de Louvain (2012/23MAR/125 and 2018/19DEC/475) and Vilnius Regional Biomedical Research Ethics Committee (No. 15820-14-743-260) approved the use of human ovarian tissue for this study.

### Ovarian tissue freezing and thawing

After obtaining informed consent, ovarian tissue biopsies from six patients (aged 25–30 years) who had undergone surgery for non-ovarian pathologies were taken for follicle isolation. For OSC isolation, ovarian biopsies were taken from five post-menopausal patients (aged 51–68 years) who had provided informed consent and undergone surgery for non-ovarian pathologies. All biopsies were cryopreserved using a conventional slow-freezing protocol (Amorim *et al.*, 2009). For the experiments, cryovials containing frozen ovarian fragments (n = 18) were thawed as described by Amorim *et al.* (2009).

### Preantral follicle isolation

Preantral follicles were isolated following the protocol described by Chiti *et al.* (2017). Briefly, ovarian tissue fragments were mechanically minced with a tissue chopper (McIlwain Tissue Chopper, Loughborough, UK) and incubated in Dulbecco’s phosphate-buffered saline (PBS) with Ca^2+^ and Mg^2+^ (Gibco, Thermo Fisher Scientific, Ghent, Belgium) supplemented with 0.28 Wünsch units/ml Liberase DH (Roche Diagnostics, Mannheim, Germany) and 12 Kunitz units/ml DNase I (Roche Diagnostics) in a water bath (37°C) for 30 min with agitation, and the cells were pipetted every 15 min. To stop the enzymatic digestion, the same volume of PBS + 10% heat-inactivated fetal bovine serum (HIFBS; Gibco, Thermo Fisher Scientific) was added after 30 min, and the suspension was filtered with a cell strainer of 70 μm (PluriSelect Life Science, Leipzig, Germany). The digestion step was repeated with ovarian tissue fragments collected from the cell strainer. Every 30 min, the filtration and follicle recovery steps were repeated until 90 min of enzymatic digestion were reached. Isolated follicles were investigated and picked up with a 130 μm micropipette (Cook, Limerick, Ireland) under a stereomicroscope (Leica DMIL, Diegem, Belgium).

### OSCs isolation and *in vitro* culture

Isolation of human OSCs was adapted from the protocol described above. Briefly, ovarian tissue pieces were mechanically minced and incubated in PBS with Ca^2+^ and Mg^2+^ supplemented with Liberase DH and DNase I in a water bath (37°C) for 75 min with agitation and pipetting every 15 min. The digestion was inactivated with the same volume of PBS + 10% HIFBS and filtered with a cell strainer of 70 μm, then 30 μm. The filtered cell solution was centrifuged at 500×*g* for 10 min at 4°C and the pellet was suspended in DMEM/F12 (Gibco, Thermo Fisher Scientific) supplemented with 10% HIFBS, 1% Antibiotic-Antimycotic (Anti-Anti; Gibco, Thermo Fisher Scientific) and 1% insulin, transferrin, and selenium (ITS; Gibco, Thermo Fisher Scientific). Cells were counted using Trypan blue (Sigma-Aldrich, Bornem, Belgium) and a Bürker chamber (VWR, Leuven, Belgium), with ∼105 viable cells being seeded per well. Cells obtained from individual patients were cultured separately *in vitro*. The time required for the cell monolayer to achieve confluence varied, ranging from 4 to 7 days depending on the patient.

### Follicle viability

To assess the viability of isolated preantral follicles, 5 to 15 follicles (freshly isolated or soon after encapsulation in the alginate hydrogel) were incubated in 50 μl of PBS containing 2 μmol/l calcein AM and 5 μmol/l ethidium homodimer-I (LIVE/DEAD^®^ Viability/Cytotoxicity Kit, Life Technologies, Amsterdam, the Netherlands) for 30 min at 37°C in the dark and then analyzed using a Zeiss LSM 510 confocal microscope. The follicles were classified into four categories (V1 to V4) depending on oocyte and granulosa cell (GC) viability (Martinez-Madrid *et al.*, 2004) (Supplementary Fig. S1): V1, live follicle with all GCs and oocyte viable; V2, minimally damaged follicle with <10% of dead GCs; V3, moderately damaged follicle with 10–50% of dead GCs; and V4, dead follicle with more than 50% of dead GCs and/or a dead oocyte. V1 and V2 were considered viable follicles; V3 and V4 were classified as damaged or dead. Data are presented as a single pool of follicles on a specific day and condition.

### Follicle encapsulation and *in vitro* culture

Follicles were *in vitro* cultured in 1% alginate hydrogel as this polymer concentration yielded favorable outcomes in terms of follicle viability and growth (Amorim *et al.*, 2009; Camboni *et al.*, 2013; Vanacker *et al.*, 2013; Chiti *et al.*, 2022). Briefly, NovaMatrix 3% SLM alginate (NovaMatrix, Sandvika, Norway) was diluted to 1.11% with 3-(N-morpholino) propanesulfonic acid buffer (MOPS, Sigma-Aldrich) (Vanacker *et al.*, 2013). All follicles were pooled in groups up to 10 (depending on total isolated follicle number: 8–10 follicles per bead) and were transferred into these alginate solution drops (to reach a final concentration of 1%), surrounded by 0.1 M CaCl_2_ to cross-link, and washed three times in MOPS for 5 min. A total of 1–3 alginate beads, depending on the number of isolated follicles (Fig. 1B), were placed into a separate well of a 4-well plate with or without an OSC monolayer and cultured in a medium containing DMEM/F12 supplemented with 10% HIFBS, 1% Anti-Anti, 1% ITS, 50 mg/ml pyruvic acid (Thermo Fisher Scientific), 0.2% Menopur (75 IU of FSH and LH; Ferring, Malmö, Sweden) and 30 ng/ml recombinant human basic fibroblast growth factor (PeproTech EC, London, UK), 50 ng/ml insulin-like growth factor 1 (IGF1; PeproTech EC), 30 ng/ml epidermal growth factor (PeproTech EC), 40 ng/ml growth differentiation factor 9 (Sigma-Aldrich), and 100 ng/ml stem cell factor (PeproTech EC). All follicles were cultured at 37°C, 5% CO_2_ for up to 7 days. Every 2–3 days, half of the culture medium was replaced.

### Follicle classification

Follicle diameter was measured at Days 0, 3, and 7 of *in vitro* culture using an inverted microscope with a calibrated eyepiece graticule. Follicles were classified according to Lierman *et al.* (2015) as primordial (≤60 µm), primary (61–75 µm), or secondary (76–200 µm). The primordial class encompasses both primordial and intermediate follicles. Moreover, secondary follicles were further classified into early/mid (75< x ≤100 µm), mid/late (100< x ≤150 µm), and late stages (150< x µm) (Kristensen *et al.*, 2011). Data are presented as a single pool of follicles on a specific day.

### Estradiol measurement using ELISA

Medium was collected from wells containing alginate beads encapsulating follicles at various stages of development. Frozen medium from Day 0 and Day 7 of cell culture were thawed for estradiol (E2) measurements using an ELISA assay (ELK1208; ELK-Biotechnology, Wuhan, China) as instructed by the manufacturer. The samples from each patient were assessed in duplicate and could be detected within the range of 15.63 to 1000 pg/ml.

### Statistical analysis

Statistical analysis was performed using GraphPad Prism, version 9.1.2 (San Diego, CA, USA). The quantitative data (diameter of follicles and E2 concentration) were reported as mean ± SD and analyzed using ANOVA and Tukey multiple comparisons *post hoc* test or Student’s *t*-test complying with normal distribution and homogeneity of variances. Qualitative data (viability and developmental stage) were analyzed using the Chi-square test or Fisher’s exact test. Differences were considered statistically significant when the *P*-value was <0.05.

## Results

### Follicle isolation

A total of 382 follicles were isolated from 18 ovarian tissue fragments. The mean diameter of the isolated follicles was 40.8 ± 9.9 µm (with 92% of the follicles ranging 30–50 µm). Most were primordial/intermediate (97%; 370/382), while only 3% were at the primary stage (12/382). Immediately after isolation, 96% of the follicles were viable (24/25) (Table 1).

**Table 1.** Follicle viability before and after *in vitro* culture.

Follicle classification	D0	D7	
		With OSC monolayer	Without OSC monolayer
**Viable (V1** **+** **V2)**	96%	87%	77%*
	(24/25)	(41/47)	(31/40)
**Damaged/dead (V3** **+** **V4)**	4%	13%	23%*
	(1/25)	(6/47)	(9/40)

OSC: ovarian stromal cells; V1/V2: live/live with minimally damaged follicles; V3/V4: moderate damaged/dead follicles. One-tailed Fisher’s exact test was used to statistically evaluate the data.

* Significant difference between D0 and D7, *P* < 0.05.

### Follicle survival after *in vitro* culture

**Figure 2.**
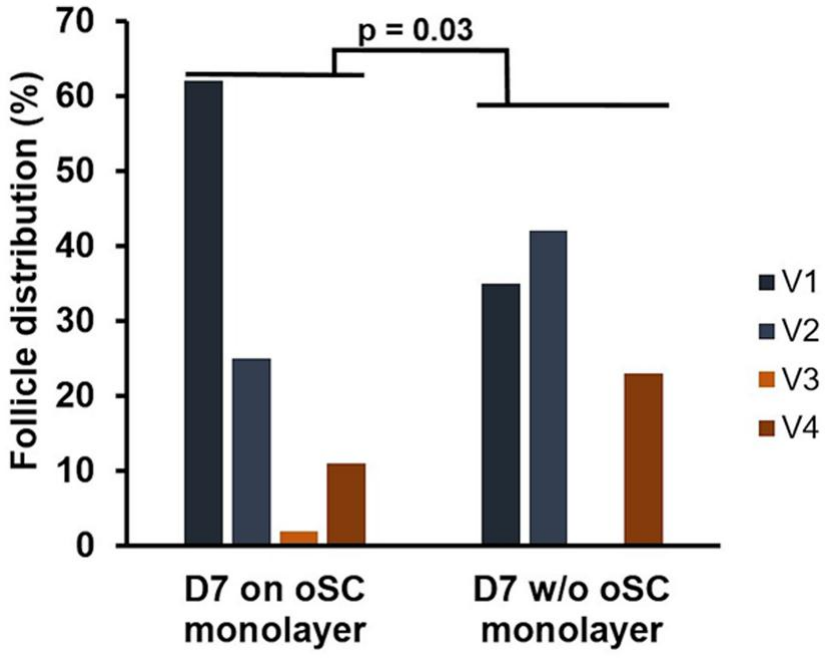
**Viability distribution of the follicles on Day 7 of *in vitro* culture on an ovarian stromal cell (OSC) monolayer or no stromal cell monolayer.** V1/V2: live/live with minimally damaged follicles; V3/V4: moderate damaged/dead follicles. Data are presented as a single pool of primordial and growing follicles: n = 47 from the OSC monolayer group and n = 40 from the group without an OSC monolayer. Two-tailed Fisher’s exact test was used to statistically evaluate the data.

After 7 days of *in vitro* culture, 87 follicles (47 from the OSC monolayer group and 40 from the group without OSC monolayer) were used for the viability assay. The analysis showed no significant difference in the proportion of viable (V1 + V2) and damaged (V3 + V4) follicles between the two groups. However, when compared to Day 0, we observed a significant decrease (*P* = 0.043) in the viability of the follicles grown without an OSC monolayer, while those grown on an OSC monolayer did not experience significant damage (*P* = 0.225) (Table 1). Interestingly, there was a significant difference (*P* = 0.03) in the distribution pattern of V1, V2, V3, and V4 follicles between the two groups on Day 7 (Fig. 2). We observed almost twice as many follicles of V1 quality grade and half as many dead (V4) follicles in the group grown on OSCs.

### Follicle development during *in vitro* culture

**Figure 3.**
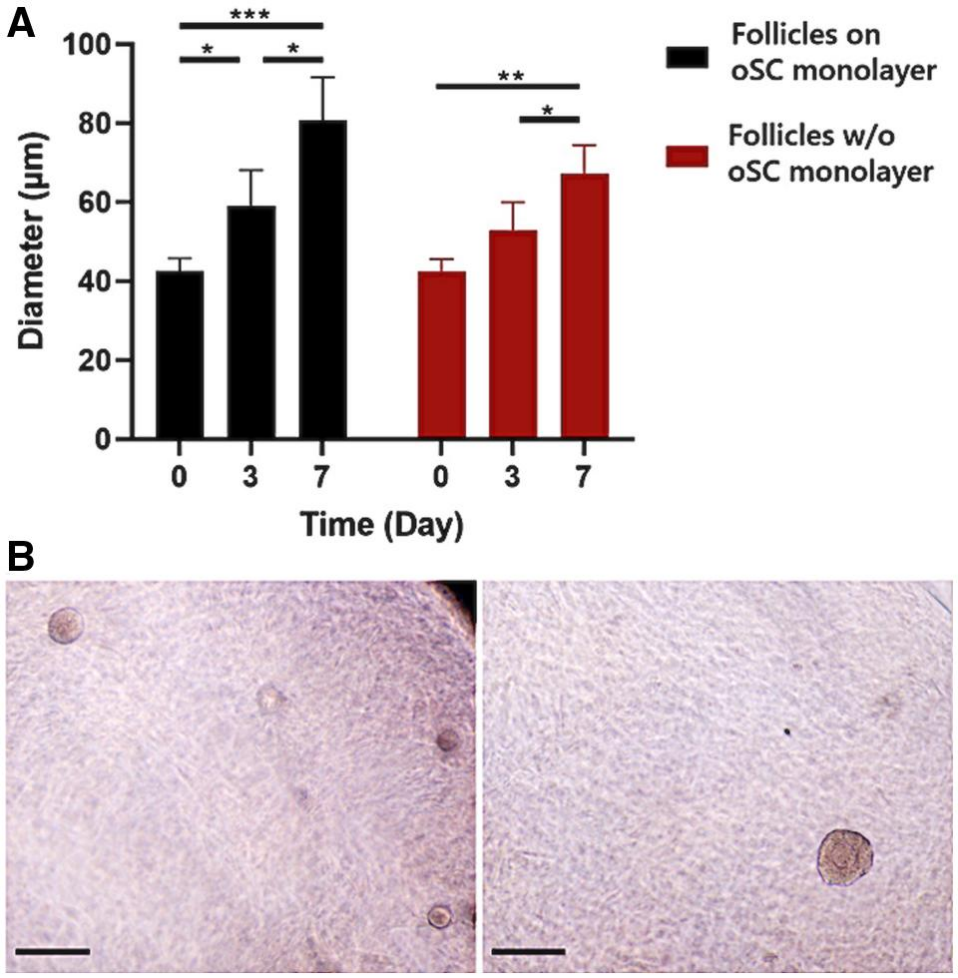
**Growth of follicles during 7 day *in vitro* culture on an ovarian stromal cell (OSC) monolayer or no stromal cell monolayer.** (**A**) Follicle diameter on Days 0, 3, and 7 of *in vitro* culture (data presented as a single pool of primordial and growing follicles). ANOVA and Tukey correction was used to statistically evaluate the data. Significant differences are indicated as follows: *P < 0.05, **P < 0.001, ***P < 0.0001. (**B**) Representative images of primordial (left image, right corner; 60 µm) and secondary (left image, left corner; 90 µm and right image; 150 µm) follicles maintaining follicular integrity in 1% alginate after 7 days of being encapsulated in groups of ∼10 follicles. Scale bar 200 µm.

The isolated follicles encapsulated in 1% alginate hydrogels were divided into two groups: 184 were *in vitro* cultured on an OSC monolayer, while 186 were *in vitro* cultured without the OSC. During culture, a significant increase in the mean diameter of the follicles was observed within both groups on different days of *in vitro* culture (Fig. 3). On Day 7, follicles on the OSC monolayer tended to grow faster and larger (80.65 ± 10.98 µm) compared to those without the OSC monolayer (67.30 ± 7.19 µm). However, we did not observe a significant difference between the two groups (*P* = 0.07). Throughout the 7 days of *in vitro* culture, we found a significant change in the distribution of primordial/intermediate and growing follicles within both groups (*P* < 0.0001) (Table 2). Nonetheless, a significantly higher number of growing follicles were found after 3 (*P* < 0.05) and 7 (*P* < 0.0001) days of *in vitro* culture with OSC monolayer compared to the group cultured without OSCs (Table 2). This suggests that, following 3 and 7 days of *in vitro* culture, a higher number of primordial/intermediate follicles progressed into primary and secondary follicles in the group treated with an OSC monolayer (Supplementary Table S1).

**Table 2.** Distribution of primordial/intermediate and growing (primary and secondary) follicles before and after *in vitro* culture.

	Day 0		Day 3		Day 7	
	Primordial/intermediate	Growing	Primordial/intermediate	Growing	Primordial/intermediate	Growing
**With OSC monolayer**	96%	4%	70%	30%^1^^,^^a^	35.5%	64.5%^2^^,^^3^^,^^c^
	(184/191)	(7/191)	(124/177)	(53/177)	(51/144)	(93/144)
**Without OSC monolayer**	97%	3%	81%	19%^1^^,^^b^	56%	44%^2^^,^^3^^,^^d^
	(186/191)	(5/191)	(141/175)	(34/175)	(84/149)	(65/149)

OSC: ovarian stromal cells. Chi-square test was used to statistically evaluate the data. Distributions of (primordial/intermediate and growing) follicles with different superscripts differ significantly between the groups on specific days:

^a,b^ *P* < 0.05;

^c,d^ *P* < 0.001, while distributions within the groups on all days (

^1^Day 0 vs Day 3;

^2^Day 0 vs Day 7;

^3^Day 3 vs D 7) had a significant difference of *P* < 0.0001.

On Day 3, we observed that 23 of the 28 secondary follicles grown on the OSC monolayer group were at the early/mid-stage and 5 were at the mid/late stage. In contrast, all 13 secondary follicles in the group without OSC were at the early/mid-stage during the same period. On Day 7, we found 46 early/mid, 16 mid/late, and 2 late (170 and 200 µm) secondary follicles after culture on the OSC monolayer, compared with 36 early/mid, 7 mid/late, and 1 late (170 µm) secondary follicle grown without OSC.

The measurement of E2 in the spent medium on Day 7 revealed that follicles at different developmental stages encapsulated in alginate beads cultured on an OSC monolayer secreted a significantly higher amount of this hormone compared to those cultured without OSC (54.1 ± 14.2 vs 29.9 ± 4.0 pg/ml; *P* = 0.006).

## Discussion

In recent years, numerous studies have explored various culture conditions aimed at fostering follicle growth *in vitro*, predominantly relying on ovarian tissue culture (Laronda *et al.*, 2014; Xiao *et al.*, 2015; Younis *et al.*, 2017; Schallmoser *et al.*, 2022). The achievement of our study is the demonstrated development of human ovarian primordial/intermediate follicles into secondary follicles within a remarkably short span of one week in a three-dimensional *in vitro* co-culture setting. This significant advancement offers a distinct advantage over cultures involving portions of the ovarian cortex, which typically contain a heterogeneous mix of preantral follicles, making it challenging to pinpoint the precise occurrence of primordial follicle activation. Nevertheless, our assessment was limited to microscopic evaluation of follicle developmental stages, grouping primordial and intermediate follicles into a single group based on established classifications (Griffin *et al.*, 2006; Lierman *et al.*, 2015) without detailed morphological scrutiny. This fusion categorizes both stages as non-growing follicles, as outlined by Gougeon *et al.* (1994). While our culture system did not enable the individual tracking of each follicle’s growth, the initial sizes of follicles in the lower developmental stage strongly indicate substantial growth to reach the higher developmental stages.

Nonetheless, our study provides a unique perspective by specifically investigating the influence of the follicle co-culture with a feeder layer of OSCs. An intriguing aspect of our research lies in the utilization of OSCs derived from post-menopausal women. This choice enables us to capture a cell population that more accurately mirrors the ovarian tissue of patients who have undergone cancer treatment. For cancer patients, the development of an artificial ovary utilizing preantral follicles isolated from cryopreserved ovarian tissue before cancer treatment, coupled with ovarian cells isolated from the ovarian tissue of patients in remission, holds significant promise. While our primary emphasis lies in addressing the fertility concerns of women and young girls facing hematological malignancies, the broader impact of this approach extends to other high-risk patient groups. Specifically, young women and prepubertal girls at risk of ovarian metastasis from neuroblastoma and Ewing sarcoma (Grubliauskaite *et al.*, 2023), stand to benefit from our innovative methodology. Furthermore, patients undergoing gonadotoxic treatment due to autoimmune diseases (Marder *et al.*, 2012) could benefit from this fertility restoration approach. This broader clinical applicability adds a layer of significance and relevance to our findings, underscoring the potential impact of our research on diverse patient populations. The stroma of premenopausal ovaries is known to comprise a diverse range of cell types, including oocytes, granulosa cells, immune system cells, endothelial and perivascular cells, as well as stromal cells, all of which remain incompletely characterized (Fan *et al.*, 2019; Kinnear *et al.*, 2020; Wagner *et al.*, 2020). In contrast, post-menopausal ovaries primarily consist of populations such as endothelial cells, immune cells, lymphatic endothelial cells, perivascular cells, smooth muscle cells, and stromal cells (Lengyel *et al.*, 2022). Notably, these aging ovaries exhibit a characteristic inflammatory environment, which has been identified as one of the mechanisms contributing to follicle depletion (Ansere *et al.*, 2021; Lliberos *et al.*, 2021; Ouni *et al.*, 2022), suggesting that they are not conducive to supporting folliculogenesis. However, cells isolated from post-menopausal ovaries have demonstrated remarkable plasticity *in vitro*, being able to survive, proliferate, differentiate, secrete hormones, and synthesize extracellular matrix (Asiabi *et al.*, 2020; Maruhashi *et al.*, 2020; Dadashzadeh *et al.*, 2022). In our study, we demonstrated that a high number of these cells can be successfully isolated from frozen and thawed ovarian tissue, and their survival and proliferative capacity are evident through the formation of a confluent cell layer within a few days of *in vitro* culture. Although a formal assessment of cell viability was not conducted at the conclusion of the experiments, it is noteworthy that cells from all patients maintained a robust and healthy morphology throughout the entire *in vitro* culture period. While our study did not involve an in-depth characterization of the specific cell populations after isolation or at the end of the study, previous analyses from our group suggest that the majority of cells isolated from post-menopausal ovarian cortex are likely fibroblasts and other mesenchymal cells, with a minimal proportion of endothelial cells (Dadashzadeh *et al.*, 2022). Furthermore, supplementation with fetal bovine serum has been shown to increase the proportion of CD73+ and CD90+ cells, potentially comprising fibroblasts, mesenchymal stem cells or pericytes, while further reducing the numbers of endothelial and endothelial progenitor cells (Dadashzadeh *et al.*, 2022). These cells secrete a myriad of growth factors known to exert significant influences on primordial follicle activation and/or preantral follicle growth through paracrine signaling. These factors include hepatocyte growth factor (Parrott *et al.*, 1994), transforming growth factor alpha (Scurry *et al.*, 1994), basic fibroblast growth factor (Almeida *et al.*, 2012), epidermal growth factor (Scurry *et al.*, 1994; Mao *et al.*, 2004), insulin-like growth factor (Monget *et al.*, 1996), tumor necrosis factor-alpha (Kaipia *et al.*, 1996), interleukin-6 (Maeda *et al.*, 2007), and vascular endothelial growth factor (Danforth *et al.*, 2003; Yang and Fortune, 2007).

Cell-feeding layers have already shown positive effects on the *in vitro* survival and/or growth of preantral follicles from mice (Tingen *et al.*, 2011; Itami *et al.*, 2011; Tagler *et al.*, 2012; Sarabadani *et al.*, 2021), cows (Itoh and Hoshi, 2000), and women (Wu *et al.*, 2021). These cells were fibroblasts from different sources, like mouse embryos (Tagler *et al.*, 2012) and skin (Itoh and Hoshi, 2000) and human dermis (Wu *et al.*, 2021) or specifically isolated from the ovaries, such as mesenchymal cells (Itoh and Hoshi, 2000), interstitial cells (Itami *et al.*, 2011), and macrophages and theca cells (Tingen *et al.*, 2011).

These studies demonstrate the interplay between cells and follicles through the synthesis of cytokines, growth factors, and hormones. Moreover, an additional evaluation of the reciprocal relationship between the follicles and OSCs, potentially explaining the follicular influence in our study of OSCs from post-menopausal women, would determine the ideal level of factor regulation essential for enhanced follicle growth and survival. To this end, it is crucial to identify the specific cells and factors contributing to the dynamics of our *in vitro* co-culture system. For this, a comprehensive analysis both before and after *in vitro* culture is indispensable. This analytical approach is essential for precisely identifying and quantifying the various cell populations that contribute to follicle survival and growth. However, identifying these cells can be a challenging task, as even advanced techniques such as single-cell RNA sequencing have not been able to fully characterize the distinct cell clusters within the ovarian stroma (Fan *et al.*, 2019). Alternatively, the identification of the bioactive factors in the spent media could be performed by enzyme-linked immunosorbent assay or liquid chromatography with tandem mass spectrometry (Chiswick *et al.*, 2012; Nakashima *et al.*, 2018) to reveal the interplay between OSCs and follicles.

In conclusion, our study demonstrated that human OSCs from post-menopausal tissue did not exert a major influence on follicle growth. Nevertheless, incorporating an OSC feeder layer, which presumably enriched the culture medium with specific growth factors and cytokines, notably enhanced the transition of non-growing follicles into functional growing follicles. Furthermore, this strategy resulted in a notably higher viability rate during the 7-day *in vitro* culture period compared to cultures without a feeder layer.

Our promising findings propel us to further investigate stage-specific follicle growth, developmental capacity, and maintenance of structural integrity. Future studies will focus on elucidating the intricate paracrine signaling pathways mediated by an optimal, yet complex variety of biomolecules derived from OSCs. Such research holds the potential to advance our understanding of follicle development and may have important implications for improving assisted reproductive technologies and fertility preservation strategies for cancer patients.

## Supplementary Material

hoad052_Supplementary_Data

## Data Availability

The data underlying this article are available in the article and in its online supplementary material.
