# Supplementary material for: Influence of ovarian stromal cells on human ovarian follicle growth in a 3D environment
Source: Hum Reprod Open. 2023 Dec 21;2024(1):hoad052. doi: 10.1093/hropen/hoad052 (PMC10776356; doi:10.1093/hropen/hoad052)
Supplement: hoad052_Supplementary_Data [file hoad052_supplementary_data.zip › HRO-23-0177-R2-SuppTable1_EO.docx]

**Supplementary Table S1. Primordial/intermediate, primary, and secondary follicle distribution before and after *in vitro* culture.**

|  | **Day 0** | | | **Day 3** | | | **Day 7** | | |
| --- | --- | --- | --- | --- | --- | --- | --- | --- | --- |
|  | **Primordial/**  **Intermediate** | **Primary** | **Secondary** | **Primordial/**  **Intermediate** | **Primary** | **Secondary** | **Primordial/**  **Intermediate** | **Primary** | **Secondary** |
| **With OSC monolayer** | 96%  (184/191) | 4%  (7/191) | 0%  (0/191) | 70%  (124/177) | 14%  (25/177) | 16%^a^  (28/177) | 35.5%  (51/144) | 20%  (29/144) | 44.5%^c^  (64/144) |
| **Without OSC monolayer** | 97%  (186/191) | 3%  (5/191) | 0%  (0/191) | 81%  (141/175) | 12%  (21/175) | 7%^b^  (13/175) | 56%  (84/149) | 14%  (21/149) | 30%^d^  (44/149) |

OSC: ovarian stromal cells. Chi-square test was used to statistically evaluate the data. Distributions (primordial/intermediate, primary and secondary) with different superscripts differ significantly between the groups on the exact day (^a,b^p < 0.05;^c,d^p < 0.001).
