# Supplementary material for: Influence of ovarian stromal cells on human ovarian follicle growth in a 3D environment
Source: Hum Reprod Open. 2023 Dec 21;2024(1):hoad052. doi: 10.1093/hropen/hoad052 (PMC10776356; doi:10.1093/hropen/hoad052)
Supplement: hoad052_Supplementary_Data [file hoad052_supplementary_data.zip › Grubliauskaite_et_al_-_HRO_-_Supplementary_Figure_S1_EO.docx]

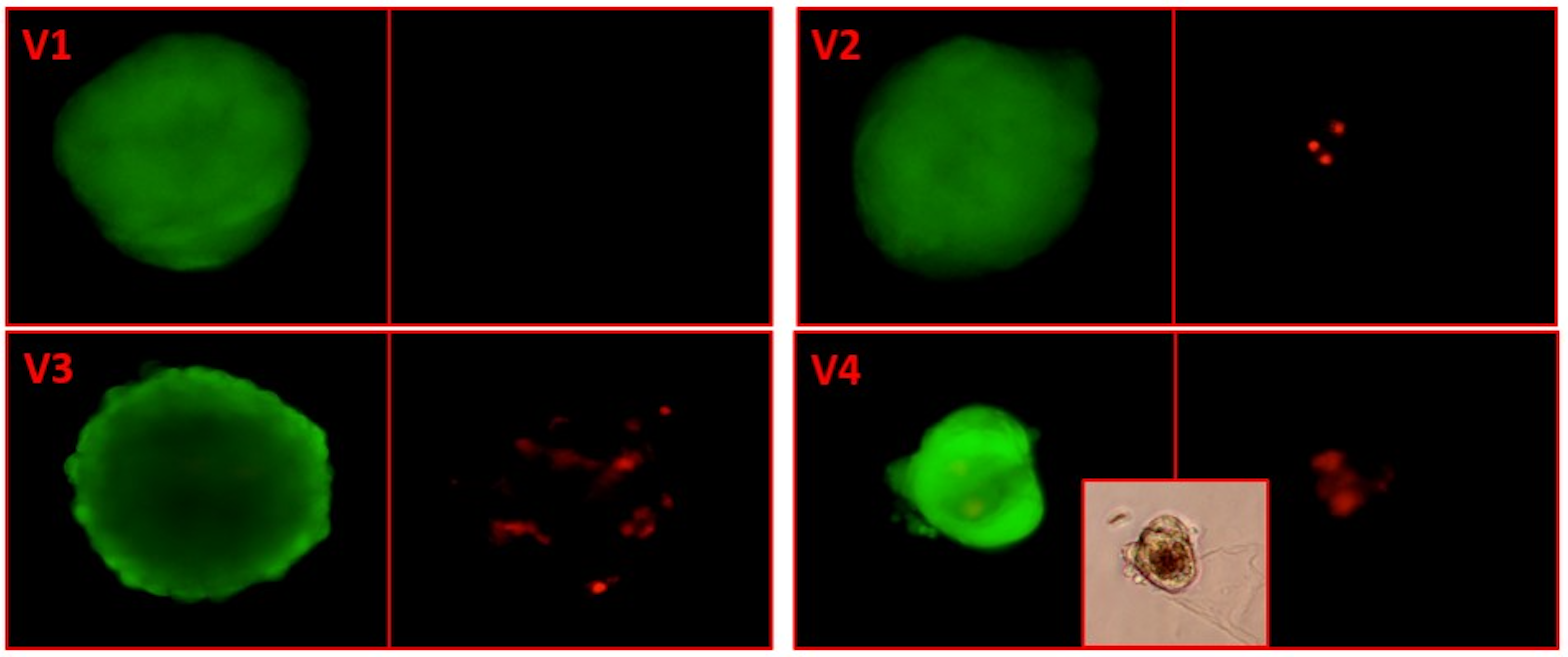


**Supplementary Figure S1. Follicle classification of the follicles according to their viability.** V1: viable follicle; V2: viable follicle with minimal damage <10% of dead granulosa cells; V3: moderate damaged follicle with 10–50% of dead granulosa cells; V4: dead follicle with >50% of granulosa cells and/or dead oocyte. Insert of bright field microscopy image shows a follicle with a dead oocyte.
